# Protective effect of snail secretion filtrate against ethanol-induced gastric ulcer in mice

**DOI:** 10.1038/s41598-021-83170-8

**Published:** 2021-02-11

**Authors:** Enrico Gugliandolo, Marika Cordaro, Roberta Fusco, Alessio Filippo Peritore, Rosalba Siracusa, Tiziana Genovese, Ramona D’Amico, Daniela Impellizzeri, Rosanna Di Paola, Salvatore Cuzzocrea, Rosalia Crupi

**Affiliations:** 1 Department of Chemical, Biological, Pharmaceutical and Environmental Sciences, University of Messina, Via F. Stagno D’alcontres 31, 98166 Messina, Italy; 2 School of Medicine, 1402 South Grand Blvd, St Louis, MO 63104 USA; 3 Department of Veterinary Science, University of Messina, Viale Annunziata, 98168 Messina, Italy

**Keywords:** Drug discovery, Gastroenterology, Health care, Molecular medicine, Medical research, Drug development, Experimental models of disease, Preclinical research, Translational research

## Abstract

Gastric ulcer or peptic ulcer is a common disease worldwide. Basically, it develops when there is an imbalance between the protective and aggressive factors, especially at the luminal surface of epithelial cells. Thus, there is a constant interest in research new drugs for treatment of gastric ulcer. The snail secretion is a dense mucous, that covers the external surface of the snails, with important functions for the survival of snails. The biological proprieties of snail *Helix Aspersa Muller* mucus it has been known for centuries to treat human disorders in particular for skin disease. Recently the use of snail mucus has seen a worldwide increase, as a component in cosmetic product and it has been used in particular for the management of wound and skin disorders. In this study we use a murine model of ethanol intragastric administration which has been widely used to test the drugs efficacies and to explore the underlying mechanism for gastric ulcer development. The intragastric ethanol administration causes several mucosal damages and an induction of a severe inflammatory response. Our results show a significant protective effect of snail secretion filtrate in reducing macroscopic and histological lesions, as well the protective effect on mucus content, oxidative stress and inflammatory response. In conclusion this study demonstrate the protective effect of intragastrical snail secretion filtrate, in a model of ethanol-induced gastric ulcer in mice, suggesting its possible useful use in the treatment or prevention of gastric ulcer.

## Introduction

The gastric ulcer or peptic ulcer is a disease that affect a significative number of people worldwide. Basically, it develops when there is an imbalance between the protective and aggressive factors at the luminal surface of epithelial cells. The most common factors contributes to the development of gastric ulcer are the *Helicobacter pylori* infection, long term use of aspirin or other NSAID, and other factors such as alcohol drinking, smoking and dietary habits^1–3^. The causes mentioned above cause an imbalance in normal mucosal barrier. In fact, normally there is a layer of adherent mucus on gastric luminal surface. Furthermore, the gastric mucus act as protective layer for the underlying epithelium, from gastric juice and pepsin. The gastric mucus content is normally balanced by the secretion of new mucus to maintain a continuous barrier, against the continuous action of gastric juice and pepsin^4^. For the treatment of gastric ulcer several drugs are available, the common approach is based on the use of H receptor antagonism and proton pump inhibitors, or the use of drugs with mechanical protective action on gastric mucosa^5^. However a prolonged use of these drugs may cause serious adverse effect^6^. Thus, there is a constant interest in research in new drugs for treatment of gastric ulcer, with a particular interest in substances of natural origin. The snail secretion or mucus is a dense mucous that covers the external surface of the snail. This mucus is produced by a particularly salivary epidermal glands in snail. The mucus has various function for the life of snails with his adhesive, emollient, protective and reparative proprieties^7^. The *Helix Aspersa Muller* mucus, is still not well characterized composition^7^. In fact, the bioactive substances present in this peculiar natural product, make it a unique product not replicable in the laboratory and with synthetic compounds. The biological proprieties of *Helix Aspersa Muller* snail mucus it has been known for centuries to treat human disorders in particular for skin disease. Recently the use of snail mucus has seen a worldwide increase, as a component in cosmetic product and it has been used in particular for the management of wound and treatment of chronic bronchitis^8,9^. It has been seen that the snail mucus components are able to stimulated the formation of dermal components, in particular the formation of collagen and elastin, and to minimized the damage generated by oxidative stress and free radicals^10^. It has shown to possess also other important biological properties such as antimicrobial activity and protective effect in wound repair^11^. In fact, the effectiveness of *Helix Aspersa* extract has been demonstrated as a safe and effective alternative treatment in open wound management of partial thickness burns in adults, favoring its re-epithelialization^9^. These important properties of snail slime and in particular pro-epithelizing and wound repair are due to the peculiar chemical composition of this compound^12^. Moreover, many of the components present in snail slime play a fundamental role in homeostasis and protection of the gastric mucosa. In particular a protective effect in the gastric mucosa has already been demonstrated for some of the components most present in the Snail Secretion Filtrate (SSF) such as, collagen, elastin, glycolic acid and allantoin^13,14^. However, there are numerous other compounds present in snail slime that contribute to its peculiar protective action, such as the mineral component^15^, due for example to the presence of copper in the SSF. In fact, copper has been shown to have an anti-ulcer action^16^. Finally, also the mucopolysaccharide component naturally present in snail slime plays a key role in the protective activity on mucous tissues^17,18^. Therefore, on the basis of the known properties of snail slime and the pathophysiology of gastric ulcer, the hypothesis of this study is that oral treatment with SSF may be a useful tool in maintaining homeostasis of the gastric mucosa, and in the prevention or treatment of inflammation of the gastric mucosa and therefore of the gastric ulcer. The murine model of intragastric ethanol administration has been widely used to test the drugs efficacies and to explore the underlying mechanism for gastric ulcer development, the intragastric ethanol administration causes several mucosal damage and an induction of a severe inflammatory response^19,20^. Alcohol also induce an important increase in oxidative stress by the generation of reactive oxygen species and lipid peroxidation, the oxidative stress play a key role in gastric ulcer disease^21^. In this study for the first time we study the potentially protective effect of Snail Secretion Filtrate in an experimental model of ethanol induced gastric ulcer in mice. Evaluating the effect on gastric mucosal, oxidative stress and inflammatory response.

## Results

### Snail secretion filtrate (SSF) chemical characterization

**Figure 1:**
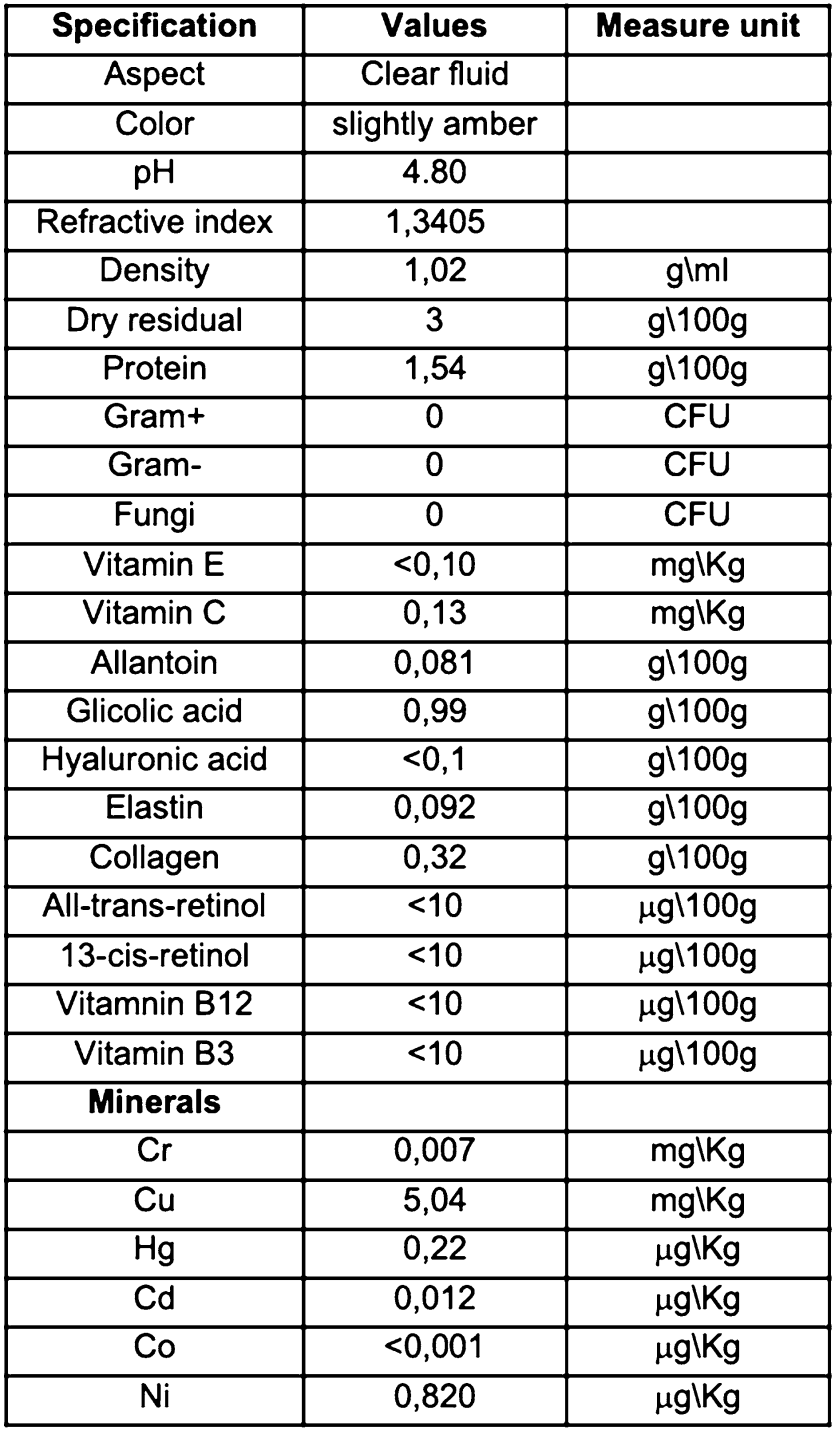
Qualitative and quantitative analysis of crude SSF. Full chemical characterization are shown in supplementary figure S1-S7.

Figure 1 panel a, reports the qualitative and quantitative properties and composition of SSF. The SSF used in this study showed a high content in glycolic acid and collagen follow by allantoin and elastin as showed by the chemical characterization showed in Fig. 1 and supplementary figure S1-S8. The dose of SSF used in this experimental model of ethanol-induced acute ulcer in mice was calculated based on maximum volume in ml/kg that can be given by o.s. in mice, so as to gradually obtain a greater surface of gastric mucosa in contact with SSF. In particular were chosen as a high dose 15 ml/kg, medium 7.5 ml/kg and low 3 ml/kg. The total protein content in SSF was 25,6 mg/ml. The Sodium dodecyl sulfate–polyacrylamide gel electrophoresis (SDS-PAGE) profiles of proteins pattern of SSF from *Helix Aspersa Muller* are shown in Supplementary Figure S1.

### Effect of SSF on macroscopic examination

**Figure 2:**
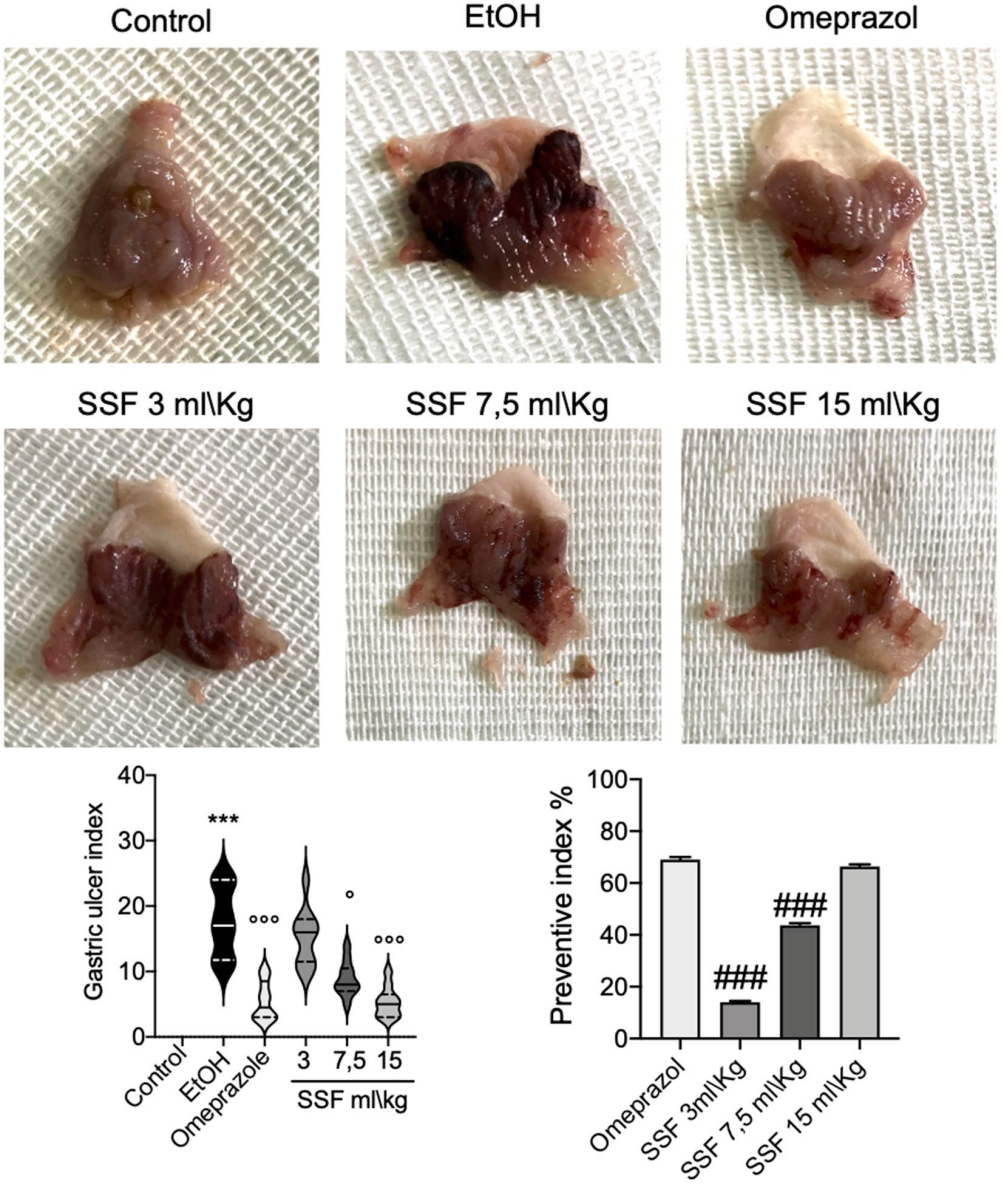
The macroscopic picture of stomach from different experimental groups. stomachs from EtOH group (the negative control group) showed a significant presence in mucosa hyperemia and mucosal damage with large ulcer formation, instead the treatment with omeprazole at dose of 20 mg/kg significant prevent the ulceration and mucosal damage induced by EtOH. The treatment with SSF showed a dose dependent protective effect. Data are presented as means ± SEM, or median with interquartile range for non-parametric data of 10 mice for each group .***p < 0.001 versus control; ◦p < 0.05 versus EtOH; ◦◦◦p < 0.001 versus EtOH; ###p < 0.001 versus omeprazole.

One hours after EtOH gastric ulcer induction, as show in Fig. 2 the stomachs from EtOH group (the negative control group) showed a significant presence in mucosa hyperemia and mucosal damage with large ulcer formation. Instead the treatment with omeprazole (used as positive control) at dose of 20 mg/kg significant prevent the ulceration and mucosal damage induced by EtOH. The treatment with SSF showed a dose dependent protective effect as showed in Fig. 2 by the graphs of gastric ulcer index and the preventive index. In particular the dose of 15 ml/kg and 7.5 ml/kg showed an important reduction in mucosal damage and ulceration compared to EtOH group.

### Effect of SSF on histological damage

**Figure 3:**
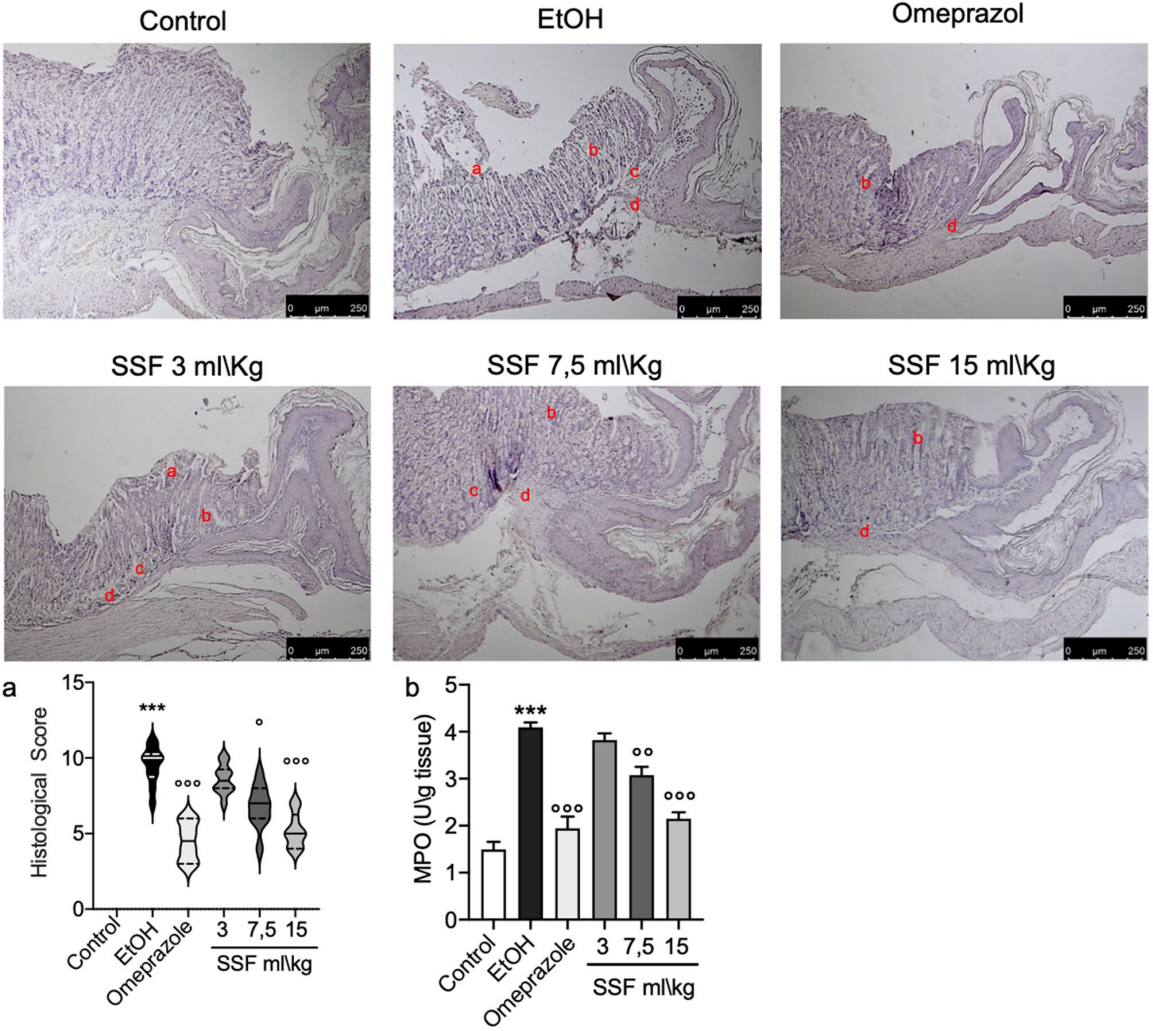
Stomach tissue section from: Control group healthy mice; EtOH group negative control; omeprazole 20 mg/kg positive control group; Histological section was assessed for: a) epithelial cell loss; b) edema in the upper mucosa; c) hemorrhagic damage; d) presence of inflammatory cells. The treatment with SSF showed a dose dependent protective effect SSF 3 ml/kg by os; SSF 7.5 ml/kg by os; SSF 15 ml/kg by os. Panel b showed the resuts of MPO assay. Data are presented as means ± SEM, or median with interquartile range for non-parametric data of 10 mice for each group.***p < 0.001 versus control; ◦p < 0.05 versus EtOH; ◦◦p < 0.01 versus EtOH; ◦◦◦p < 0.001 versus EtOH.

As show in Fig. 3a, compared to stomach tissue from control group the EtOH group showed a significant increase in tissue damaged characterized by a significant epithelial cell loss, edema, hemorrhagic damage and abundant presence of inflammatory cells . The treatment with SSF showed a dose dependent protective effect in particular with a significant protective effect on epithelial cell loss. Lower magnification of histological images on both upper and lower mucosa are shown in supplementary figure S8-S13. The presence of inflammatory cells has been confirmed thought MPO assay that showed a significant increase in MPO in EtOH group compared to control group. As show in Fig. 3b, the treatment with omeprazole significant prevent the increase in MPO, for the SSF treatment only the dose of 7.5 ml/kg and 15 ml/kg showed a significant protective effect on increase in MPO induced by EtOH.

### Effect of SSF on mucosa glycoproteins and collagen contents

**Figure 4:**
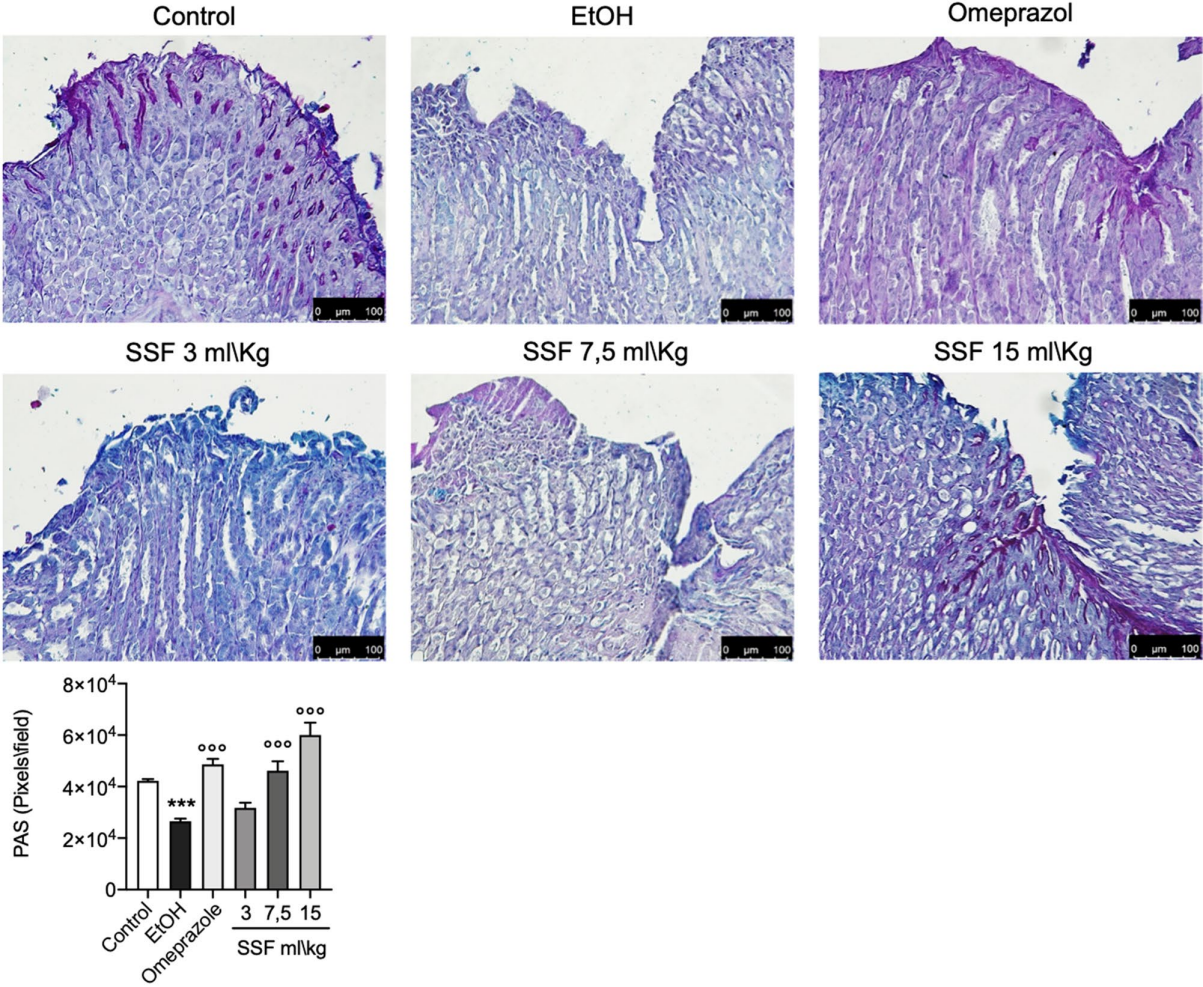
PAS staining from control group showed the normal contents of mucosa glycoprotein(magenta color); EtOH group showed a significantly reduction in PAS staining, significantly inhibited by treatment wit omeprazole 20 mg/Kg; SSF treatment showed a dose dependent protective effect as showed by the increase of PAS staining. Image J software (1.49v, https://imagej.nih.gov/ij/, 1997–2018. Schneider, C.A., Rasband, W.S., Eliceiri, K.W.) was used for the quantification of glycoprotein as the positively stained area (pixel/field). Data are presented as means ± SEM of 10 mice for each group.***p < 0.001 versus control; ◦◦◦p < 0.001 versus EtOH.

**Figure 5:**
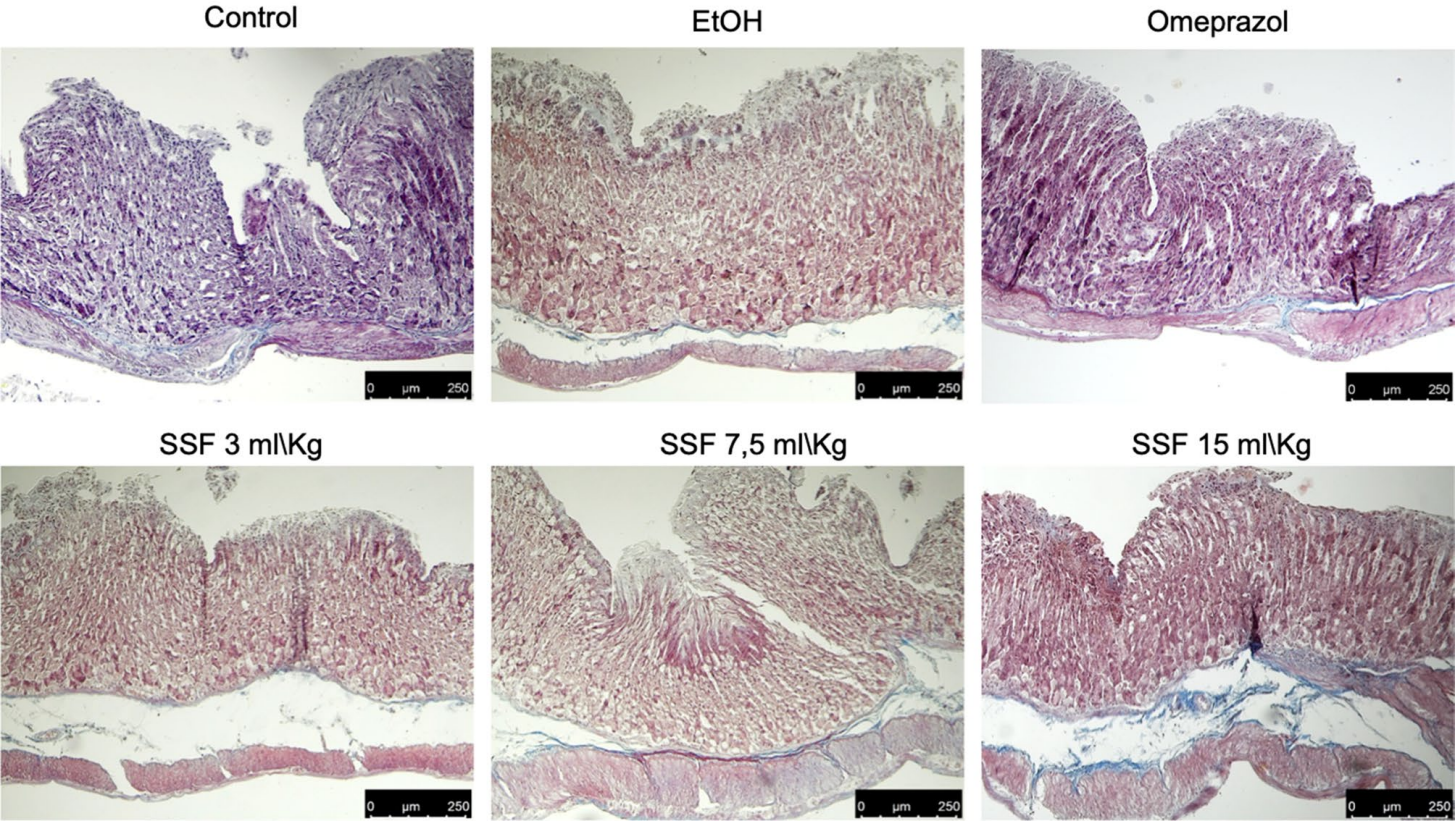
Representative image of collagen within mucosa layer using Masson’s trichrome staining. Control group showed the staining in healthy gastric tissue. EtOH group stain indicated fragmented and disorganized collagen fibers, while omeprazole group showed a significative protective effect. For the SSF treatment only the dose of 15 ml/Kg showed a significant protective effect.

Mucosa glycoprotein evaluation was performed using PAS staining. As show in Fig. 4, one hour after the EtOH administration, in the stomach from mice of EtOH group there was a significant decrease in PAS staining compared to sham group. The loss in mucosa glycoprotein was significantly reduced by pre-treatment with omeprazole. Compared to EtOH group the treatment with SSF at dose of 3 ml/Kg did not show any notable protective effect, instead the dose of 7.5 and 15 ml/kg of SSF showed a significant protective effect in a dose dependent manner compared to the EtOH group. Through Masson staining collagen can be dyed in blue, by which we can roughly evaluate the healing effect at the macro level and stains also showed the formation of granulation tissue ad collagen disorganization in the gastric wall. As show in Fig. 5, in the EtOH group stain indicated fragmented and disorganized collagen fibers, while omeprazole group showed a significative protective effect. For the SSF treatment only the dose of 15 ml/Kg showed a significant protective effect.

### Effect of SSF on mucus contents and oxidative stress

**Figure 6:**
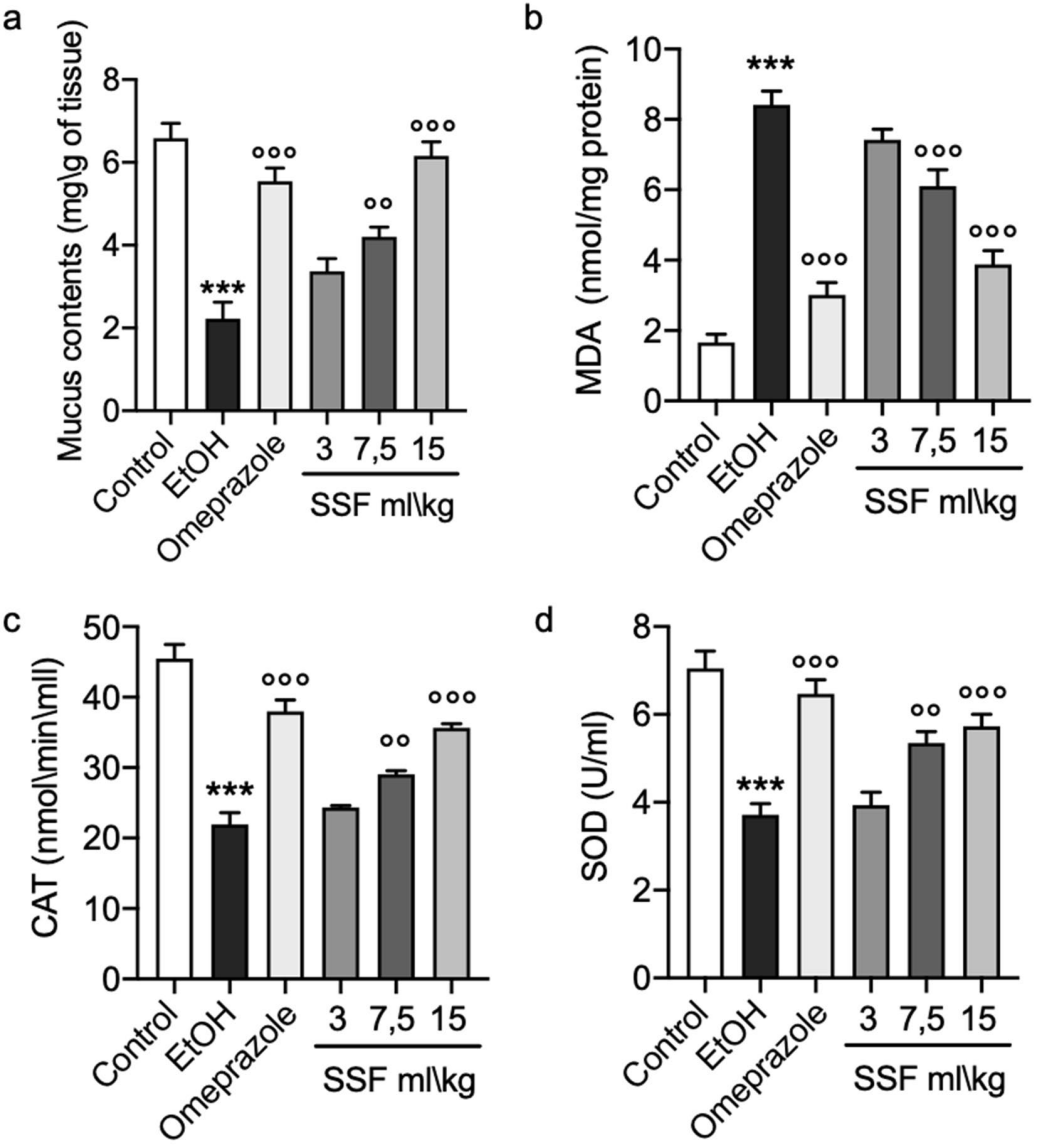
(**a**) The graph showed the mucus contents evaluation performed by alcian blue binding assay. (**b**) MDA assay levels. (**c**, **d**) CAT and SOD determination. Data are presented as means ± SEM of 10 mice for each group; .***p < 0.001 versus control; ◦◦p < 0.01 versus EtOH; ◦◦◦p < 0.001 versus EtOH;

The mucus contents assay was performed by alcian blue binding assay. As showed by the graph in Fig. 6a, the mucus contents was significantly reduced in the EtOH group compared to the control group. When compared to EtOH group, the omeprazole group showed a significant decrease in mucus contents loss, the treatment with SSF showed a dose dependent protective effect on mucus contents loss due to EtOH administration. To assess the effect of on SSF on oxidative stress we evaluated the levels of MDA as index of lipid peroxidation, as show in Fig. 6b intragastrical EtOH administration induced a significant increase MDA levels, while the treatment with omeprazole prevent this increase in MDA in a significant manner, the treatment with SSF showed a dose dependent protective effect on mucus. Next we observed a significant reduction in CAT and SOD induced by EtOH administration that has been significantly antagonized by omeprazole pre-treatment. As show in Fig. 6c,d treatment with SSF at the dose of 3 ml/kg did not demonstrate a significant protective effect instead the doses of 7.5 ml/kg and 15 ml/kg showed a significant protective effect in a dose dependent manner.

### Effect of SSF on PGE_2_ and inflammatory response

**Figure 7:**
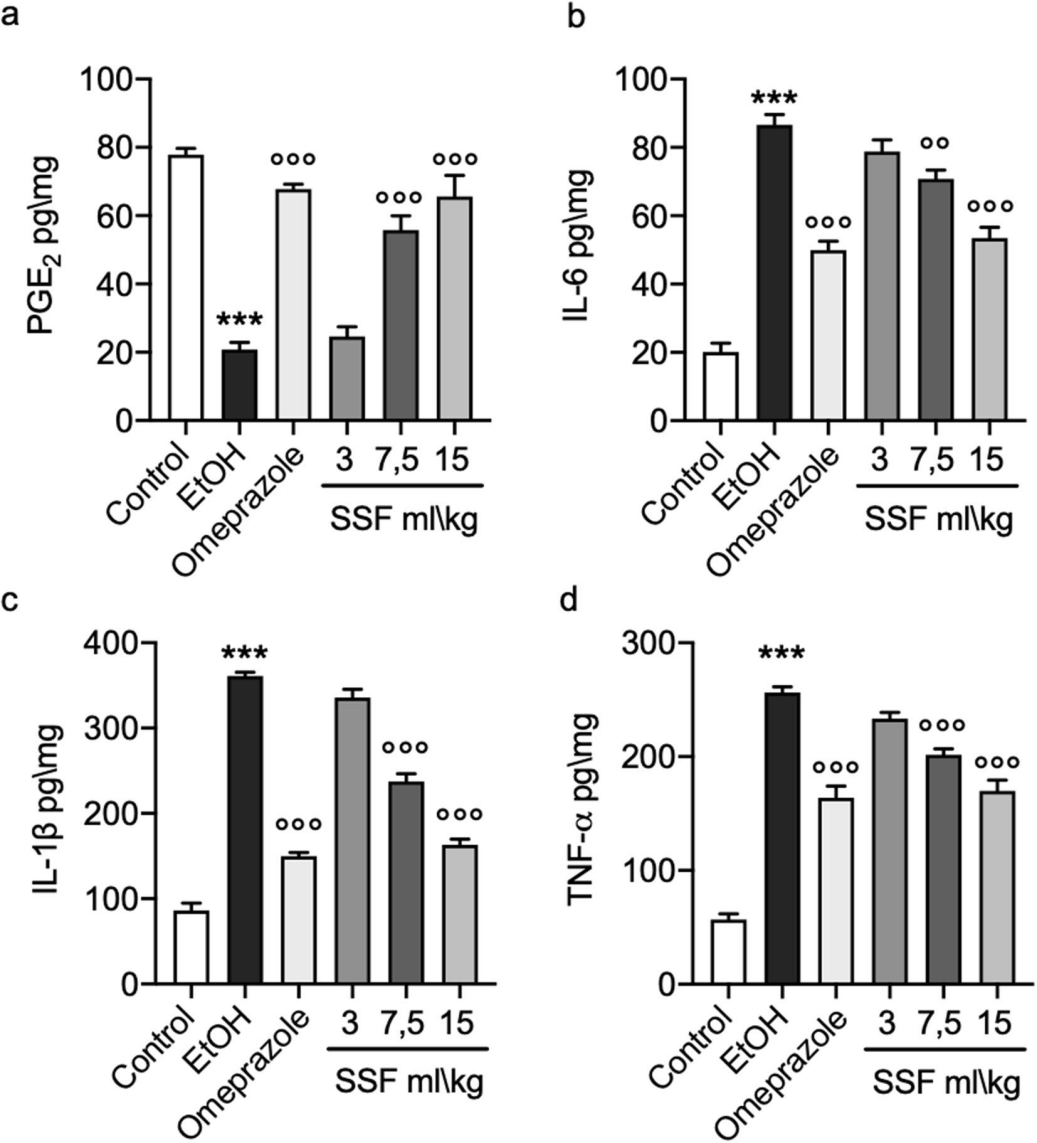
ELISA assays for (**a**) PEG_2_; (**b**) IL-6; (**c**) IL-1β; (**d**) TNF-α . Data are presented as means ± SEM of 10 mice for each group; ***p < 0.001 versus control; ◦◦p < 0.01 versus EtOH; ◦◦◦p < 0.001 versus EtOH;

As show in Fig. 7a, through ELISA assay we evaluated the levels PGE_2_ in gastric tissue homogenates, compared to control group EtOH administration induced a significant decrease in PGE_2,_ significantly antagonized by omeprazole pre-treatment. The treatment with SSF at the dose of 3 ml/kg did not demonstrate a significant protective effect instead the doses of 7.5 ml/kg and 15 ml/kg showed a significant protective effect in a dose dependent manner. Figure 7b–d, showed the results for the ELISA assays for mayor pro inflammatory cytokines IL-6, IL-1β, TNF-α, respectively. EtOH administration induced a significant increase in IL-6, IL-1β, TNF-α levels significantly antagonized by omeprazole pre-treatment. The treatment SSF at the dose of 3 ml/kg did not demonstrate a significant protective effect instead the doses of 7.5 ml/kg and 15 ml/kg showed a significant protective effect in a dose dependent manner.

## Discussion

Several factors contributes to gastric ulcer development, such as drug abuse, alcoholism, endocrine dyscrasia and bacterial helicobacter pylori infection, which can cause mucosal barrier damage through alteration in gastric acid secretion and characterized by hemorrhage and severe inflammatory response^22,23^. A key event in gastric ulcer formation is the imbalance in the gastric mucosa between the defensive factor such as mucin, prostaglandin, bicarbonate nitric oxide and growth factor, and the offensive factor such as increased secretion of pepsin and gastric acid. Thus inflammatory response, oxidative stress, and neutrophilic infiltration has been show a key role in pathophysiology of gastric ulcer, on the other hand endogenous antioxidant, mucus layer acts as protective agents^24,25^. The common approach in treating the gastric ulcer is based on H receptor antagonism and proton pump inhibitors^5^. These class of drugs may cause serious adverse effect when used for prolonged time^6^. Recently, several studies has highlighted the ability of snail secretion (or snail mucus), to improve skin conditions thanks to its peculiar proprieties such as emollient, moisturizing, lubricating and protective, especially to prevent skin damage, and thus there is a growing interest in the use of this compound in cosmetology^26^. Furthermore the *Helix Aspersa Muller* secretion has shown to possess other important biological properties such as, antimicrobial activity^11^ and wound repair^27^. Several studies have highlighted the absence of cytotoxic effects in different cell lines such as in human keratinocytes, human dermal fibroblasts (MRC-5) and murine embryo fibroblasts (NIH-3T3)^12,27^. These effect of Snail mucus is due also to his peculiar mechanical proprieties due mucopolysaccharide contents, on the other hand these effects are due to the peculiar contents of active molecules^12^. As previously demonstrated, the two molecules the glycolic acid and allantoin, most present in snail slime were believed to be an essential component for the biological activities of snail slime^9^, however recently has been demonstrated that the mucus *in toto* has a greater effect than that of the individual molecules^27^. Thus, a synergism in activity of several molecules present in snail secretion cannot be excluded, factor that make a snail secretion a peculiar compound with important biological proprieties. In fact, beyond all molecules contained in snail secretion also the specific ratio of these components in the natural snail secretion is a key factor for the biological activity. So, in this study we consider the crude Snail Secretion Filtrate (SSF) form *Helix Aspersa Muller* as compound “*in toto*” and it is not possible to understand which one the molecule is responsible for the biological activity. In this study due to the route of administration and the maximum volume in ml/kg that can be given by o.s. in mice^28^, we consider three dose of SSF as high dose of 15 ml/kg, medium of 7.5 ml/kg and low 3 ml/kg, to gradually obtain a greater surface of gastric mucosa in contact with SSF^21^. Considering the proteins content in the SSF the respective dosage as mg/kg were respectively 384 mg/kg, 192 mg/kg and 76,8 mg/kg. We choose an experimental model of ethanol intragastric administration in mice, that has been widely used to explore the underlying mechanism for gastric ulcer development and thus to test the efficacy of new drugs. The intragastric administration of ethanol cause several mucosa damage and an induction of a severe inflammatory response and oxidative stress^19–21^. In our study, the results of macroscopic gastric evaluation after EtOH administration showed significant protective effect of SSF on gastric mucosa. In particular, the treatment SSF at the dose of 3 ml/kg did not demonstrate a significant protective effect instead the doses of 7.5 ml/kg and 15 ml/kg showed a significant protective effect in a dose dependent manner. Also, on histological evaluation of gastric mucosa we confirmed the protective effect of SSF, in agreement with macroscopic score. A fundamental step in involved in the complex event of gastric ulcer formation is the infiltration of neutrophil, in particular activated neutrophil are responsible for increase in oxidative stress and inflammatory response^29,30^. Our results on MPO evaluation as index of inflammatory cell infiltration, showed that doses of 7.5 ml/kg and 15 ml/kg showed a significant protective effect in a dose dependent manner on neutrophil infiltration. A standard goal in treating gastric ulcer is preventing the decrease or degradation of gastric mucus, that act as a natural protective agent on gastric mucosa^31^. Gastric mucus consist of mucin type glycoproteins and the depletion in gastric wall mucus it has been seen to be closely related to the gastric ulcer pathology^32,33^. EtOH induced gastric ulcer is also responsible for the reduction of collagen within gastric tissue^34^. Therefore, we evaluated both the presence of glycoproteins (PAS staining) and collagen (Masson staining) following the administration of EtOH, and our results show that this has produced a significant reduction in both cases, while the treatment with SSF at doses of 7.5 ml/kg and 15 ml/kg showed a significant protective effect. These results are also confirmed by the results of mucus content performed by Alcian blue staining. According to the results mentioned so far we have found that the treatment with SSF at doses of 7.5 ml/kg and 15 ml/kg showed a significant protective effect on oxidative stress induced by EtOH, and in particular SSF treatment significantly prevent the increase in MDA levels, and the depletion in CAT and SOD levels. Oxidative stress in related to both initial stage of gastric ulcer development and with the worsening of the pathology^35^. Has previously demonstrated the gastric mucosa homeostasis is regulated by both the control of oxidative stress status and by secretion of several mediators such as cytokines and prostaglandin. In particular, prostaglandin play a key role on the state of health of the mucosa by regulating several factors such as the stimulation of mucus and bicarbonate secretion, maintaining the integrity of epithelial cells and improving mucosal mucosal blood flow, in particular PGE_2_ showed an important role in regulation of these function, an thus PGE_2_ act as a gastro protective agent^36,37^. Gastric ulcer and an gastric ulcer healing has been seen previously are regulated by the levels of prostaglandin an PGE_2_ in particular^38^. Our results indicate that gastric ulcer induced by EtOH is related with a significantly reduction on PGE_2_ levels, the treatment SSF at the dose of 3 ml/kg did not demonstrate a significant protective effect instead the doses of 7.5 ml/kg and 15 ml/kg showed a significant protective effect in a dose dependent manner. Inflammatory response is responsible for the progressive trigger and worsening of the gastric ulcer^39^. Then we evaluated the levels of the mayor inflammatory cytokines responsible for driving the inflammatory response in gastric ulcer such as IL-1β, IL-6 and TNF-α^40^. Ours results showed a significant increased levels of these cytokines in EtOH group while the treatment SSF at the dose of 3 ml/kg did not demonstrate a significant protective effect instead the doses of 7.5 ml/kg and 15 ml/kg showed a significant protective effect in a dose dependent manner in reducing the IL-1β, IL-6 and TNF-α levels. In conclusion this study demonstrate for the first time the protective effect of intragastrical snail secretion filtrate, in a model of ethanol-induced gastric ulcer in mice. The gastric cytoprotective action of snail secretion filtrate might be attributed to an increase or preservation of gastric mucus with preservation of mucosal mucopolysaccharides and collagen that which are also reflected in a reduction of oxidative stress and induced inflammatory response. surely future studies are necessary to clarify the mechanism of action of this peculiar compound, however the proven beneficial effects suggest its possible useful use in the treatment or prevention of gastric ulcer.

## Methods

### Snail secretion filtrate (SSF) collection and sterilization

Helix aspersa muller mucus was kindly provided by Snail S.R.L.S (Messina, Italy). Briefly the breeding is cruelty free. In particular the mucus was obtained mechanically manually by stimulating snails by sterile cotton swab tip. The mucus is filtered in a first step with a coarse filter stabilizing the pH, after this phase the mucus is passed in a filtration train consisting of 3 different filters (10 micron-1micron -0.22-micron, Pall) and then stored at 4 °C. The use of the 0.22 micron filter is necessary to eliminate impurities and endotoxins in order to make the product injectable.

### Snail secretion filtrate (SSF) chemical characterization

The crude Snail Secretion Filtrate extract from different batches, was qualitative and quantitative analyzed by professional service for Snail S.R.L.S (Messina, Italy), (Science4Life srl, Messina Italy. and Sialab srl. Avola, Italy) using standard analytical techniques such as IR/UV vis spectrometry and HPLC analysis to evaluate the protein quality and the allantoin and glycolic acid content respectively, and to obtain a qualitative determination of the total protein content and to a Bradford assay (Bio-Rad) to evaluate the protein amount. Elastin and collagen were analyzed as seen previously^41,42^.All-trans-retinol, 13-cis-retinol,Vitamnin B12,Vitamin B3 were analyzed according EN 12,823–1:2014, ISO20634:2015 and UNI EN 15,652:2009 respectively. All slime doses in experiments were subsequently calculated on microgram of proteins per milliliter (mg/mL) content.

### Animals

CD1 mice (male, 20–30 g; Envigo) were accommodated in a standard location (room 22 °C and 12-h light/dark cycles) with standard rodent chow and water ad libitum. The animals were adapted to these conditions for 1 week. Messina University Review Board for the care of animals approved the research. All animal experiments agree with the new regulations in Italy (D.Lgs 2014/26), EU regulations (EU Directive 2010/63). All experimental were conducted in according to ARRIVE guidelines.

### Ethanol-induced acute ulcer

Ethanol-induced acute ulcer in mice were performed as seen previously^21,43^.

Briefly the mice were fasted for 24 h and divided into the follow group:

- Control (n = 10): mice were treated by oral gavage with distilled water (15 ml/Kg)
- EtOH (n = 10): mice were treated by oral gavage with distilled water (15 ml/Kg) 1 h before ethanol-induced acute ulcer
- Omeprazole 20 mg/Kg: mice were treated by oral gavage with omeprazole at 20 mg/kg 1 h before ethanol-induced acute ulcer
- SSF 3 ml/Kg: mice were treated by oral gavage with Snail Secretion Filtrate (SSF) at 3 ml/kg 1 h before ethanol-induced acute ulcer
- SSF 7.5 ml/kg: mice were treated by oral gavage with Snail Secretion Filtrate (SSF) at 7.5 before ethanol-induced acute ulcer
- SSF 15 ml/Kg: mice were treated by oral gavage with Snail Secretion Filtrate (SSF) at 15 ml/kg 1 h before ethanol-induced acute ulcer

One hour after the oral treatment, acute ulcer was induced by orally EtOH (98% ethanol containing 150 mM HCl) administration at 5 ml/kg. Control mice were treated with an equal volume of distilled water instead of EtOH solution. Animals were euthanized 1 h after treatment with the EtOH mixture, under isoflurane inhalation anesthesia (5% in air. Baxter) by cervical dislocation. In this study due to the route of administration and maximum volume in ml/kg that can be given by o.s. in mice^21,28^, we consider three dose of Snail Secretion Filtration (SSF) as high dose of 15 ml/kg, medium of 7.5 ml/kg and low 3 ml/kg, consider the proteins content in the SSF the respective dosage as mg/kg were respectively 384 mg/kg, 192 mg/kg and 76.8 mg/kg.

### The gastric ulcer index and preventive index

After sacrificing the animals, the stomachs were quickly removed and washed with 0.9% saline. This was followed by macroscopic examination of the stomach for the detection of any hemorrhagic lesions on the glandular mucosa. The length in mm of each lesion was measured to determine the mean ulcer index (UI)^44^. The length (mm) and the width (mm) of each band was measured by Vernier caliper. The degree of gastric mucosal lesions was evaluated from digital pictures, and the severity of mucosal lesions was scored as follows: no ulcer (0 mm), 1–5 petechiae (< 1 mm) (1), 6–10 petechiae (< 1 mm) (2), > 10 petechiae (< 1 mm) (3), small linear ulcer (< 2 mm) (2), medium linear ulcer (2–4 mm) (3), and large linear ulcer (> 4 mm) (4). If the width was > 1 mm, then the points were multi- plied by two. The GU index (UI) was determined by adding the sum of the total of the scores and divided by the number of animals.The preventive index (PI) of pretreatments against ulceration was calculated according to the following equation:

$$ {\text{PI}} = \left( {{\text{UIethanol}} - {\text{UIpretreated}}} \right){\text{/UIethanol}} \times 100. $$

### Histological analysis

For histological assessment, the stomach was fixed in 10% (V/V) neutral buffered formalin solution, and then embedded in paraffin, sliced into 5 μm thicknesses, stained with hematoxylin–eosin (H&E)^45^. The specimens were examined under an optical microscope (DM5500, Leica), and were assessed according to what has seen previously^46^. Histological section was assessed for epithelial cell loss (score: 0–3), edema in the upper mucosa (score: 0–4), hemorrhagic damage (score: 0–4), and the presence of inflammatory cells (score: 0–3), yielding a maximum total score of 14. The sections were assessed by an experienced pathologist who was blinded to the study.

### Measurement of mucosa glycoproteins and collagen

To further evaluate the mucosal lesions in the gastric tissues, we performed mucosa glycoprotein measurements with periodic acid-Schiff (PAS) staining according to the manufacturer's instructions (Bio-optica, Italy). The positive glycoprotein site will appear as magenta color. Image J software was used for the determination of the positively stained area (pixel/field)^47,48^.Masson trichrome staining were performed according to the manufacturer’s protocol (Bio-Optica, Milan, Italy).

### Measurement of gastric mucus contents

Alcian blue (Sigma-Aldrich) binding assay was performed according to what seen previosly^49^. After removing the stomach, some parts of the stomach were weighted and immersed in 10 mL of 0.02% Alcian blue and 0.16 M sucrose/0.05 M sodium acetate solution (pH 5.8) and incubated at 25 °C for 24 h. The Alcian blue binding extract was centrifuged at 3000 × g for 10 min at 4 °C. The absorbance of the supernatant was measured at 620 nm on a spectrophotometer. Free mucus in the gastric content was calculated based on the amount of Alcian blue binding to the gastric mucus (mg/g of tissue).

### Myeloperoxidase activity

Gastric tissues, were homogenized and MPO activity was detected^50,51^.

### Determination of malondialdehyde (MDA) level

Thiobarbituric. acid–reactant substances measurement was determined as a marker of lipid peroxidation. Thiobarbituric acid–reactant substances were calculated by comparison the O.D. to a standard mixture of 1,1,3,3-tetramethoxypropan/99% malondialdehyde bis (dymethylacetal)/99% (MDA) (Sigma, Milan, Italy). The absorbance of the supernatant was measured spectrophotometrically at 532 nm as seen previously^52^.

### Determination of oxidative stress markers and biochemical determinations

SOD activity in the gastric tissue was determined using the Superoxide Dismutase Assay Kit (Cayman Chemicals) according to manufacturer’s protocols. CAT activity in the gastric tissue was determined using the Catalase Assay Kit (Cayman Chemicals) according to manufacturer’s protocols. The supernatant of stomach tissue homogenate was subjected to measurement of PGE2 level using an ELISA kit (Cayman chemicals) according to manufacturer protocols. IL-1β, IL-6, TNF-α were measured using ELISA kit (Invitrogen, Thermo Fisher) according to manufacturer protocols.

### Analysis of protein pattern

Analysis of protein pattern were performed as previously seen^53^. Total protein concentrations in extracts were determined by the Bradford method with bovine serum albumin (BSA) as the standard. Then, SDS-PAGE gel electrophoresis was performed on samples with equalized concentrations of total protein. In Particular 20 μl of samples was mixed with 20 μl of Laemmli Sample Buffer with β-mercaptoethanol (Bio-Rad) and heating (95 °C, 5 min). then 40 μl of mixed samples and 5 μl of protein marker (Precision Plus Protein Dual Color Standards, Bio-Rad) were loaded onto the gel and resolved, using the Mini-PROTEAN electrophoresis system (Bio-Rad). The protein bands, separated on gel, were fixed, stained in QC Colloidal Coomassie Stain and destained solution according to the producer procedure (Bio-Rad).

### Statistical analysis

All values are shown as the mean ± standard error of the mean (SEM) of N observations (N = 10) or median with interquartile range for non-parametric data. Data were analyzed by Non-parametric Kruskal–Wallis one-way ANOVA followed by Dunn's multiple comparisons test, or one-way ANOVA followed by a Bonferroni post hoc test for multiple comparisons. A p-value of less than 0.05 was considered significant: *p < 0.05 versus control; ◦p < 0.05 versus EtOH; **p < 0.01 versus control; ◦◦p < 0.01 versus EtOH; ***p < 0.001 versus control; ◦◦◦p < 0.001 versus EtOH; #p < 0.05 versus omeprazole; ##p < 0.01 versus omeprazole; ###p < 0.001 versus omeprazole.

## Supplementary Information

Supplementary Information 1.

## Data Availability

All data generated or analyzed during this study are included in this published article.
