# Supplementary material for: Protective effect of snail secretion filtrate against ethanol-induced gastric ulcer in mice
Source: Sci Rep. 2021 Feb 11;11:3638. doi: 10.1038/s41598-021-83170-8 (PMC7878904; doi:10.1038/s41598-021-83170-8)

Protective effect of snail secretion filtrate against ethanol-induced gastric ulcer in mice

Enrico Gugliandolo[3°], Marika Cordaro[1°], Roberta Fusco[1°], Alessio Filippo Peritore[1], Rosalba Siracusa[1], Tiziana Genovese[1], Ramona D'Amico[1] ,Daniela Impellizzeri[1], Rosanna Di Paola[1*], Salvatore Cuzzocrea[1,2]* and Rosalia Crupi[3]

[1]University of Messina, Department of Chemical, Biological, Pharmaceutical and Environmental Sciences , Via F. Stagno D'alcontres 31, Messina, Italy , 98166.

[2]School of Medicine, 1402 South Grand Blvd, St Louis, MO 63104, USA.

[3] University of Messina, Department of Veterinary Science, viale Annunziata, Messina, Italy, 98168.

°The authors equally contributed to this work

***Corresponding Author**
Prof. Salvatore Cuzzocrea - Department of Chemical, Biological, Pharmacological and Environmental Sciences, University of Messina, Viale F. Stagno D'Alcontres, 31 – 98166 Messina, Italy; Phone: +390906765208; email: salvator@unime.it

Prof. Rosanna Di Paola - Department of Chemical, Biological, Pharmacological and Environmental Sciences, University of Messina, Viale F. Stagno D'Alcontres, 31 – 98166 Messina, Italy; Phone: +390906765208; email: dipaolar@unime.it

Supplementary Figure 1 S1- SDS-PAGE (10%) analysis of proteins in SSF samples

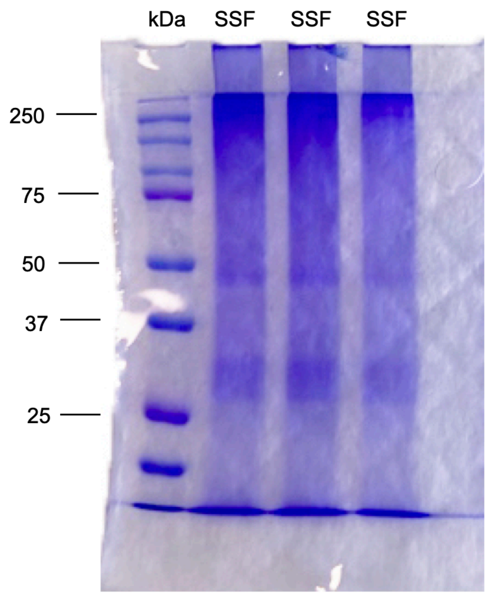

Supplementary Figure 2, S2- SSF chemical characterization, allantoin

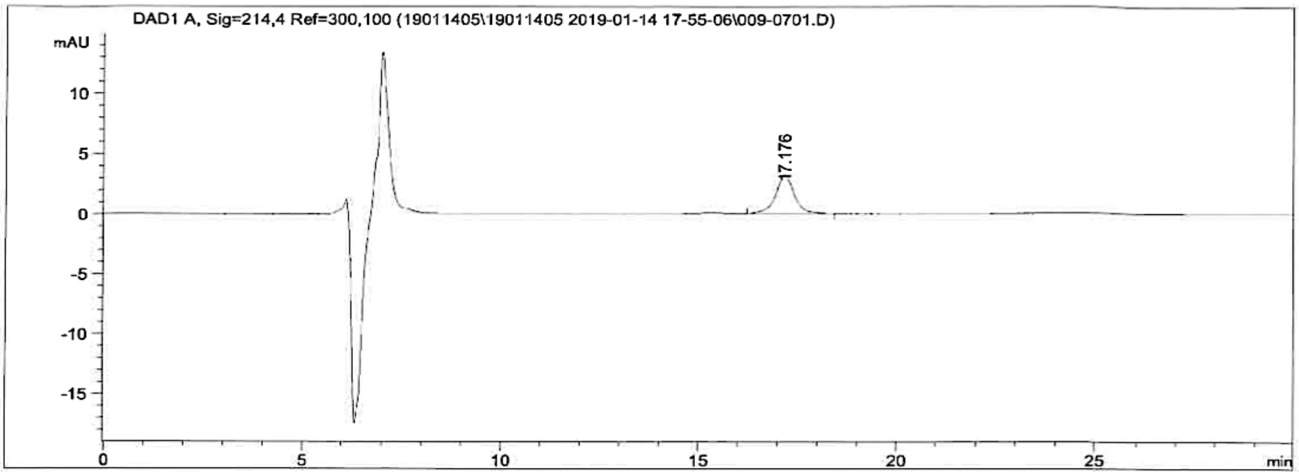


```
=====================================================================
                     External Standard Report
=====================================================================

Sorted By             :      Signal
Calib. Data Modified  :      14-Jan-19 4:04:48 PM
Multiplier            :      1.0000
Dilution              :      1.0000
Use Multiplier & Dilution Factor with ISTDs

Signal 1: DAD1 A, Sig=214,4 Ref=300,100

RetTime  Type     Area      Amt/Area     Amount    Grp   Name
 [min]          [mAU*s]                  [mg/l]
-------|------|----------|----------|----------|--|------------------
  17.176 VV    3283.91699  29.77674   8.108430        Allantoina
```

Supplementary Figure 3, S3- SSF chemical characterization, glycolic acid

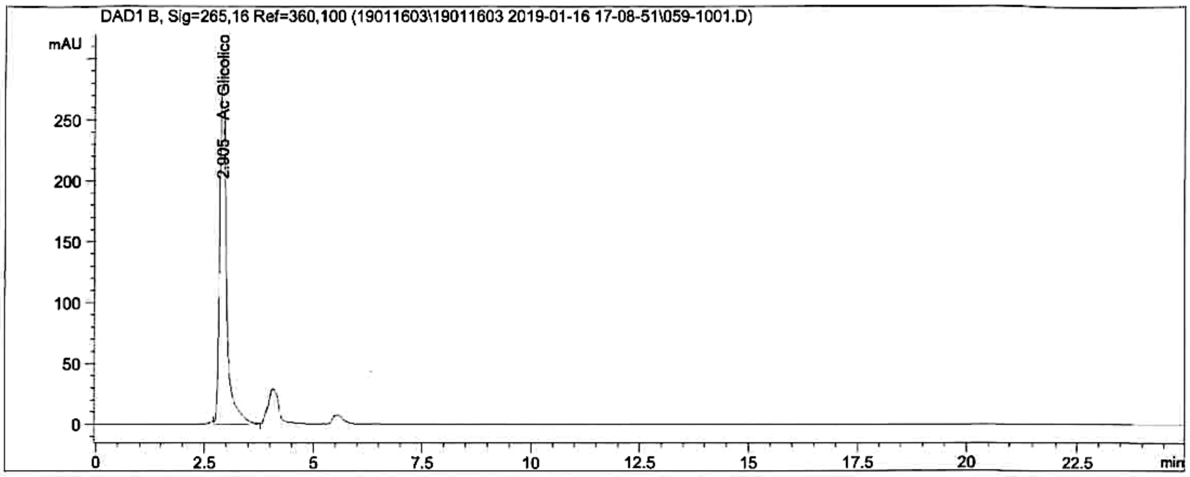


```
=====================================================================
                    External Standard Report
=====================================================================

Sorted By              :      Signal
Calib. Data Modified   :      16-Jan-19 5:15:42 PM
Multiplier             :      1.0000
Dilution               :      1.0000
Use Multiplier & Dilution Factor with ISTDs

Signal 1: DAD1 B, Sig=265,16 Ref=360,100

RetTime  Type     Area      Amt/Area    Amount   Grp   Name
 [min]          [mAU*s]               [ng/ul]
-------|------|----------|----------|----------|--|------------------
  2.905 VV    3283.91699   29.77674  99.08430     Ac Glicolico
```

Supplementary Figure 4, S4- SSF chemical characterization, aminoacidic composition

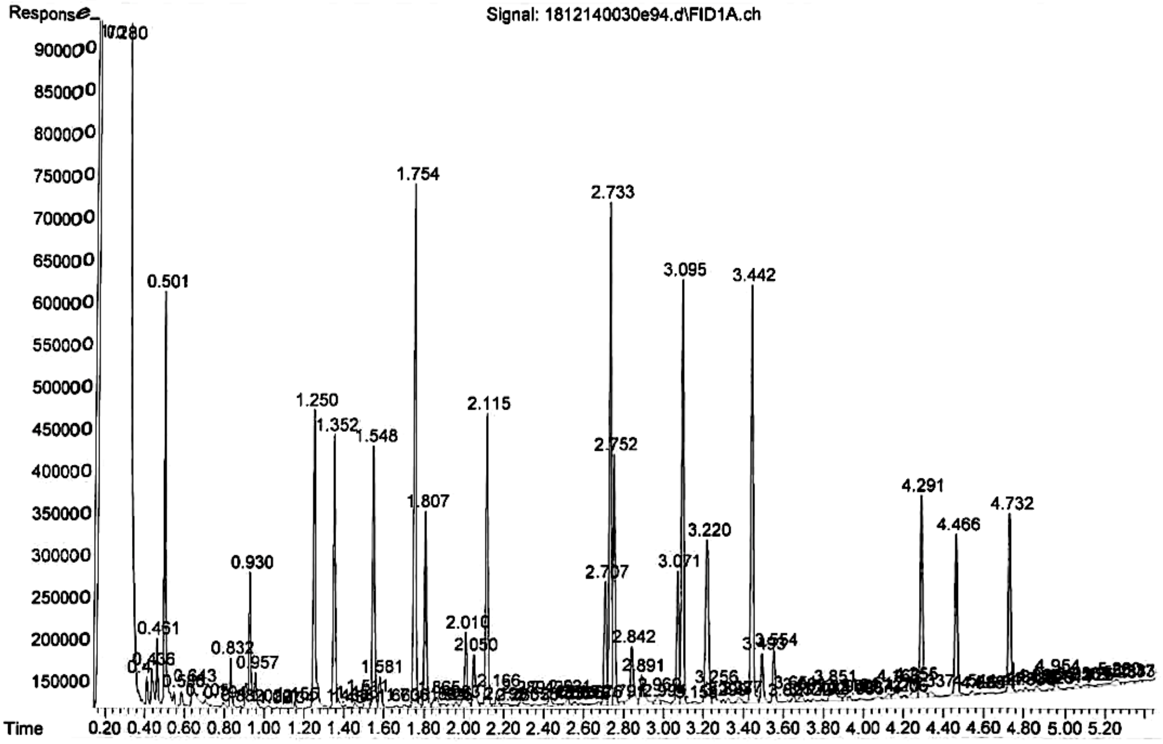

Supplementary Figure 5, S5- SSF chemical characterization, vitamin A

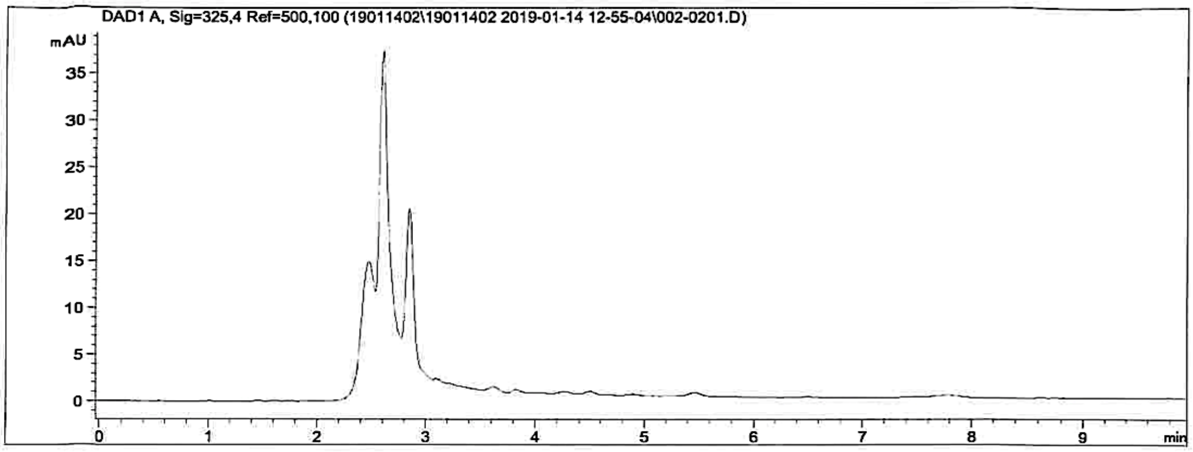


```
=================================================================
                    External Standard Report
=================================================================

Sorted By              :      Signal
Calib. Data Modified   :      Monday, Jan 14, 2019 12:54:59 PM
Multiplier             :      1.0000
Dilution               :      1.0000
Use Multiplier & Dilution Factor with ISTDs

Signal 1: DAD1 A, Sig=325,4 Ref=500,100


Signal 2: FLD1 A, Ex=325, Em=475   not found

RetTime  Type     Area      Amt/Area     Amount   Grp   Name
 [min]                                   [mg/l]
-------|------|----------|----------|----------|--|------------------
  6.076            -          -          -          cis-retinolo
  7.086            -          -          -          all-trans retinolo
```

Supplementary Figure 6, S6- SSF chemical characterization, vitamin B12

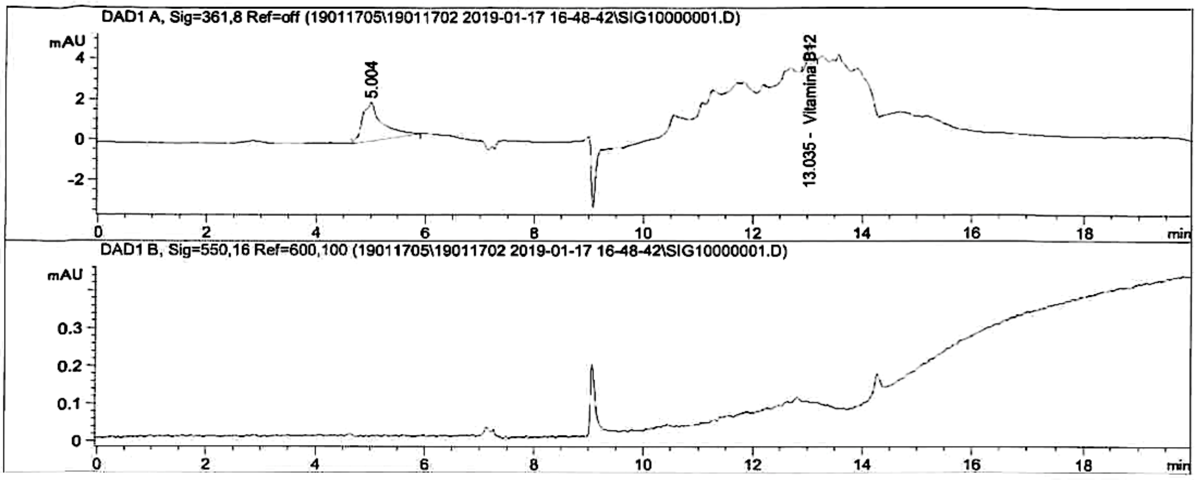


```
=====================================================================
                     External Standard Report
=====================================================================

Sorted By             :      Signal
Calib. Data Modified  :      Friday, January 18, 2019 9:00:33 AM
Multiplier            :      1.0000
Dilution              :      1.0000
Use Multiplier & Dilution Factor with ISTDs

Signal 1: DAD1 A, Sig=361,8 Ref=off

RetTime  Type    Area      Amt/Area     Amount   Grp   Name
 [min]         [mAU*s]                 [ng/ul]
-------|------|----------|----------|----------|--|------------------
 13.035 BB       6.91162    0.00000    0.00000     Vitamina B12
```

Supplementary Figure 7, S7- SSF chemical characterization, vitamin B3

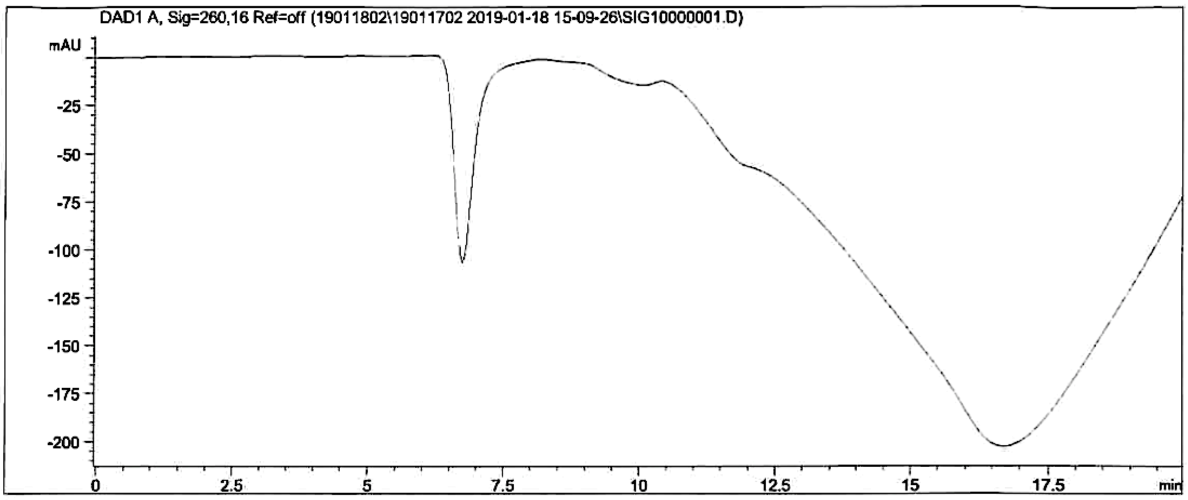


```
=====================================================================
                    External Standard Report
=====================================================================

Sorted By              :      Signal
Calib. Data Modified   :      18-Jan-19 1:10:09 PM
Multiplier             :      1.0000
Dilution               :      1.0000
Use Multiplier & Dilution Factor with ISTDs

Signal 1: DAD1 A, Sig=260,16 Ref=off

RetTime  Type    Area     Amt/Area    Amount   Grp   Name
 [min]         [mAU*s]               [ng/ul]
-------|------|----------|----------|----------|--|------------------
 11.711           -          -          -         Vitamina B3
```

Supplementary Figure 8, S8 - H&E staining of stomach tissue from SHAM group. Left side 10x magnification, on right side 40x magnification on both upper mucosa and lower mucosa respectively . Histological section was assessed for: a) epithelial cell loss; b) edema in the upper mucosa; c) hemorrhagic damage; d) presence of inflammatory cells

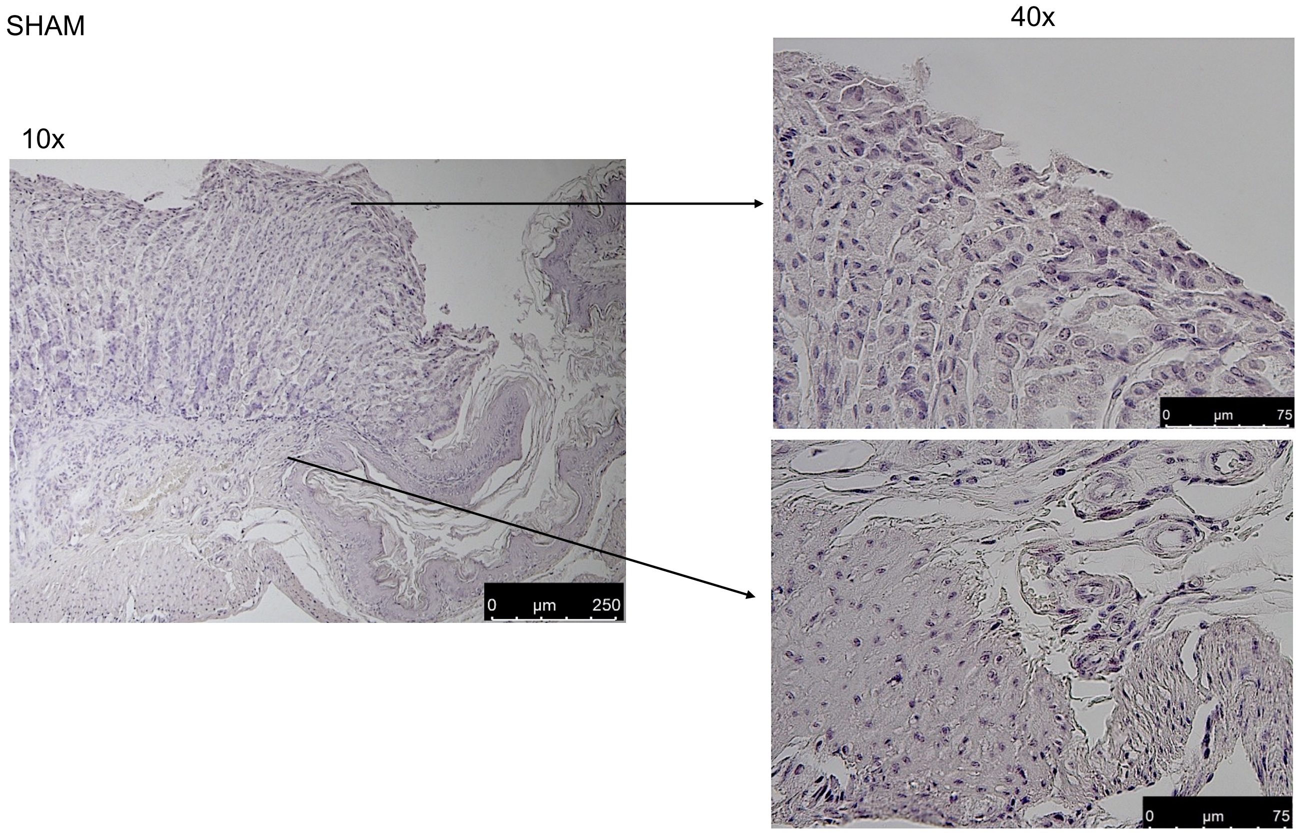

Supplementary Figure 9, S9 - H&E staining of stomach tissue from EtOH group. Left side 10x magnification, on right side 40x magnification on both upper mucosa and lower mucosa respectively . Histological section was assessed for: a) epithelial cell loss; b) edema in the upper mucosa; c) hemorrhagic damage; d) presence of inflammatory cells

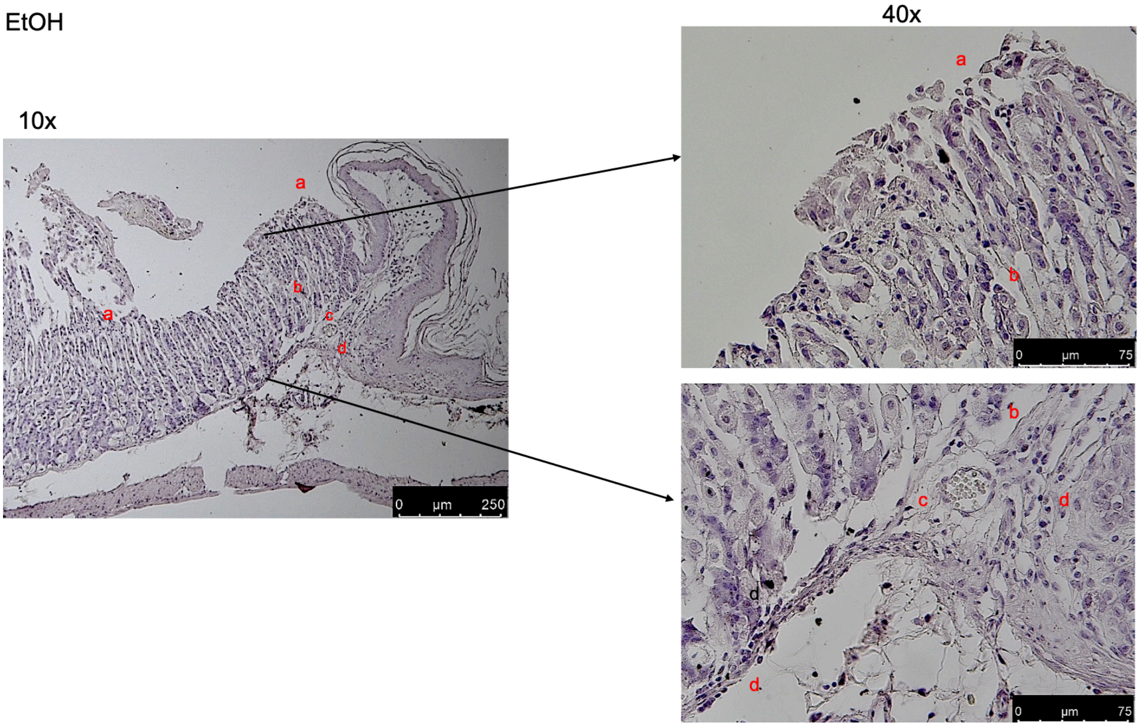

Supplementary Figure 10, S10 - H&E staining of stomach tissue from Omeprazol group. Left side 10x magnification, on right side 40x magnification on both upper mucosa and lower mucosa respectively . Histological section was assessed for: a) epithelial cell loss; b) edema in the upper mucosa; c) hemorrhagic damage; d) presence of inflammatory cells

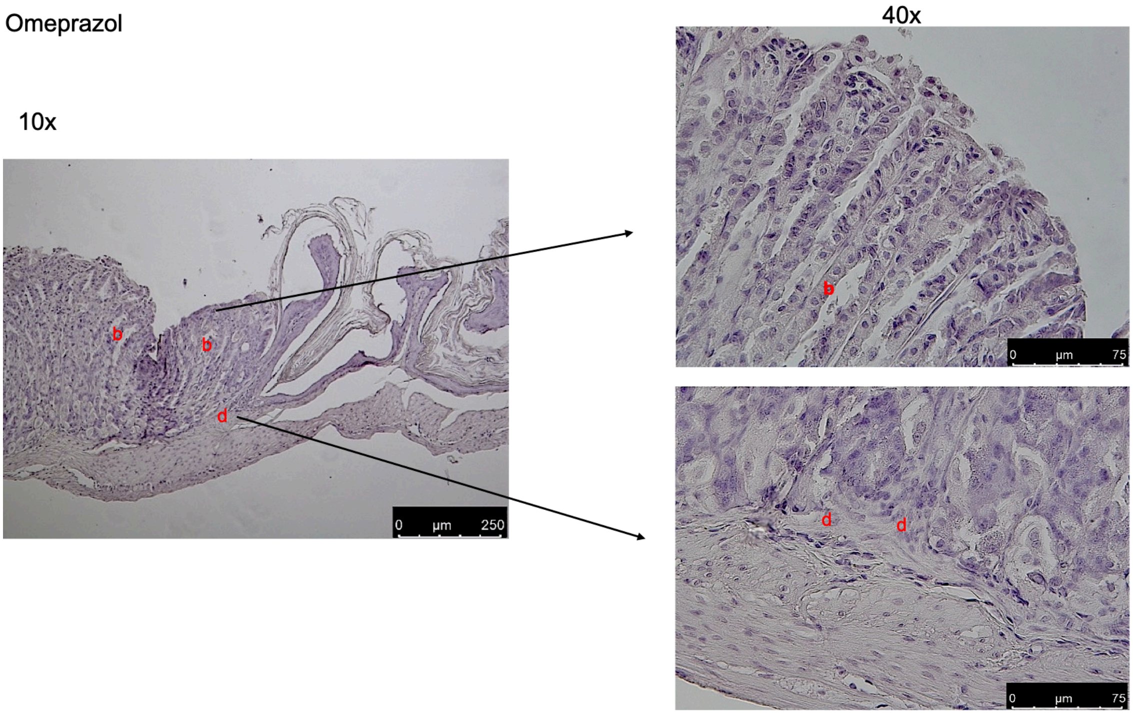

Supplementary Figure 11, S11 - H&E staining of stomach tissue from SSF 3 ml\kg group. Left side 10x magnification, on right side 40x magnification on both upper mucosa and lower mucosa respectively . Histological section was assessed for: a) epithelial cell loss; b) edema in the upper mucosa; c) hemorrhagic damage; d) presence of inflammatory cells

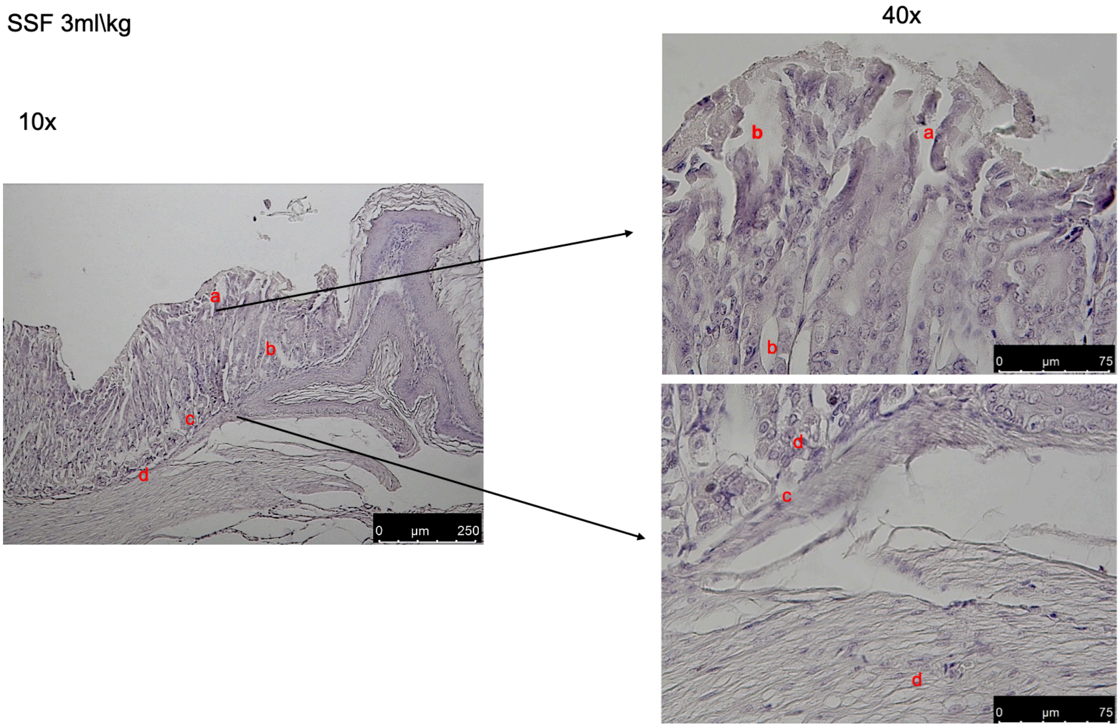

Supplementary Figure 12, S12 - H&E staining of stomach tissue from SSF 7,5 ml\kg group. Left side 10x magnification, on right side 40x magnification on both upper mucosa and lower mucosa respectively . Histological section was assessed for: a) epithelial cell loss; b) edema in the upper mucosa; c) hemorrhagic damage; d) presence of inflammatory cells

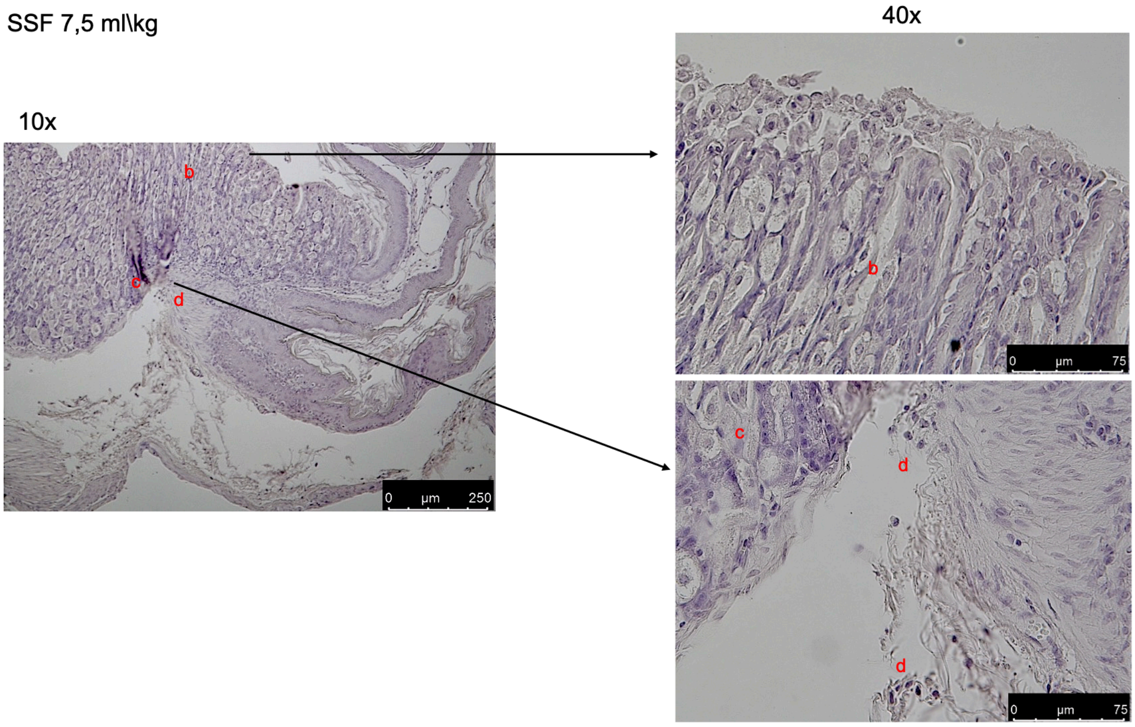

Supplementary Figure 13, S13 - H&E staining of stomach tissue from SSF 15 ml\kg group. Left side 10x magnification, on right side 40x magnification on both upper mucosa and lower mucosa respectively . Histological section was assessed for: a) epithelial cell loss; b) edema in the upper mucosa; c) hemorrhagic damage; d) presence of inflammatory cells

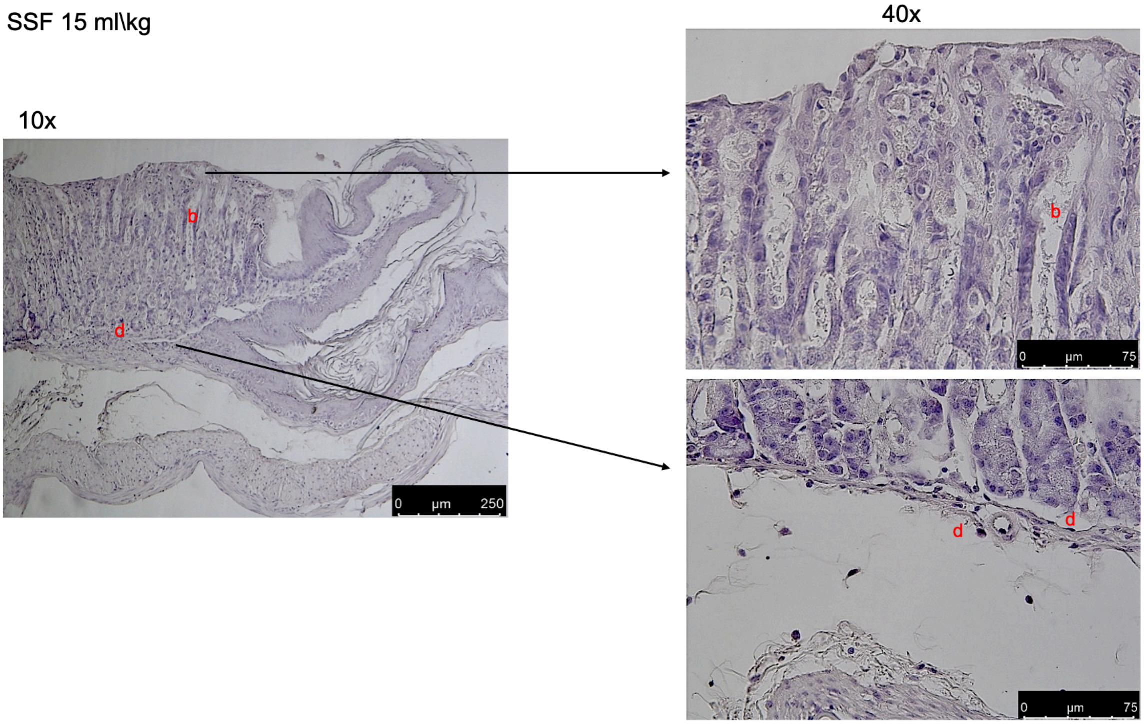

Supplement: Supplementary file 1 — Supplementary Information 1. [file 41598_2021_83170_MOESM1_ESM.pdf]
